# Endovascular treatment for dural arteriovenous fistulas in the petroclival region

**DOI:** 10.7150/ijms.47365

**Published:** 2020-10-18

**Authors:** Kun Hou, Xianli Lv, Lai Qu, Yunbao Guo, Kan Xu, Jinlu Yu

**Affiliations:** 1 Department of Neurosurgery, The First Hospital of Jilin University, Changchun, 130021, China; 2 Department of Neurosurgery, Tsinghua Changgung Hospital of Tsinghua University, Beijing 102218, China; 3 Department of Intensive Care Unit, The First Hospital of Jilin University, Changchun, 130021, China; Kun Hou and Xianli Lv contribute equally to this manuscript and they are co-first authors.

**Keywords:** Dural arteriovenous fistula, Petroclival region, Inferior petrosal sinus, Superior petrosal sinus, Endovascular treatment

## Abstract

Petroclival region dural arteriovenous fistulas (DAVFs) are rare and difficult lesions to manage. They often have very complex anatomical structures and can be further divided into the superior petrosal sinus, petrous apex, inferior petrosal sinus, upper clival, and upper clival epidural-osseous DAVFs. Most petroclival region DAVFs should be treated due to their high Cognard grades. Currently, endovascular treatment (EVT) has become the first-line therapeutic option for petroclival region DAVFs. But not all the petroclival region DAVFs could be cured with EVT. When the arterial feeders are large or the DAVF is adjacent to the venous sinus, the success rate may be higher. In petroclival region DAVFs, if EVT can be performed successfully, satisfactory outcome can be anticipated. However, there are some inadvertent complications, which include cranial nerve palsy, subsequent sinus thrombosis, and migration embolization of the internal carotid artery and vertebral artery. Currently, a review of the EVT of petroclival region DAVFs is lacking. In this article, we performed a review of the relevant literature on this issue. In addition, some illustrative cases would be provided to elaborate these specific entities.

## Introduction

**Figure 1:**
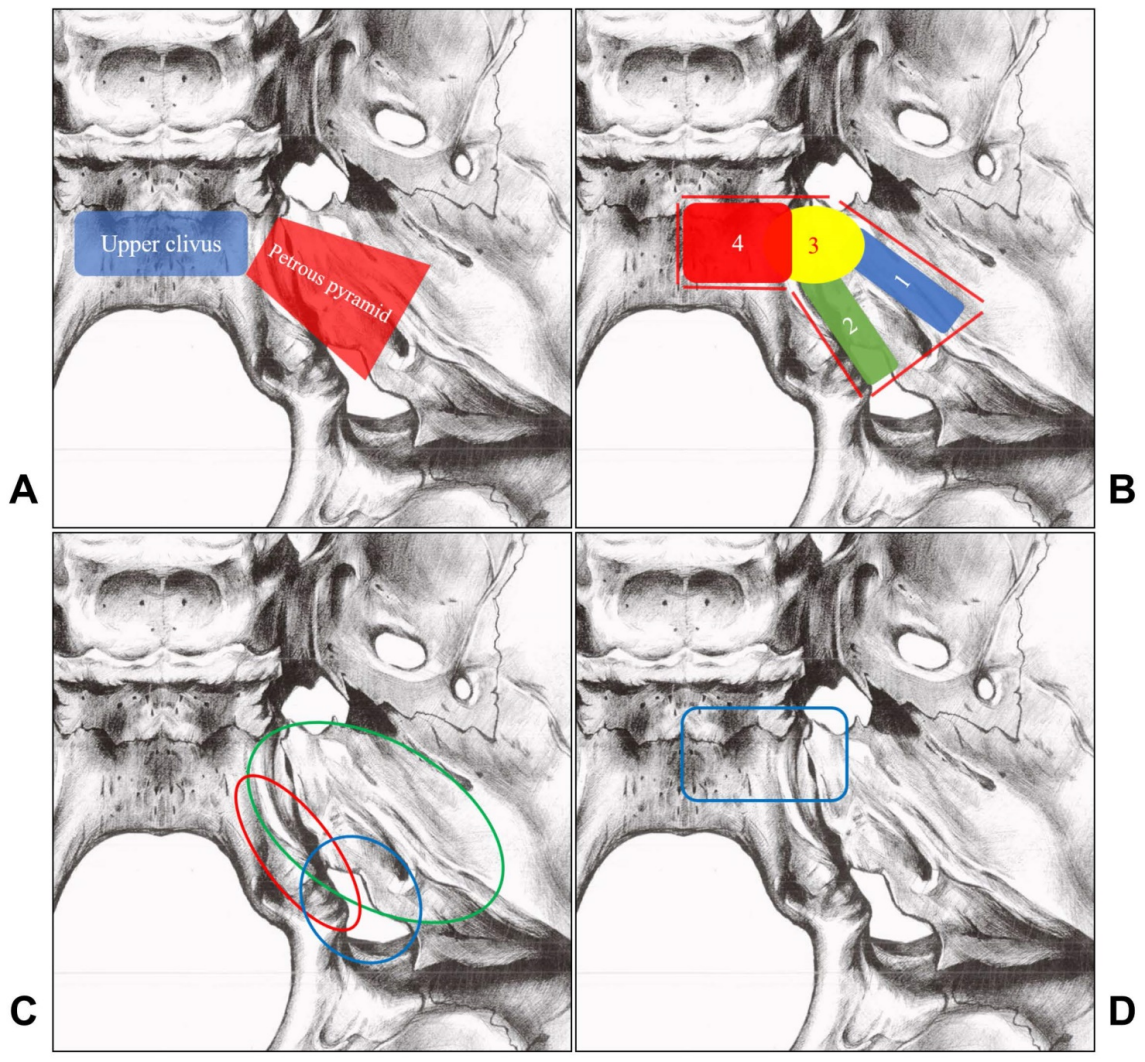
A, The petroclival region mainly includes the upper clivus (blue rectangle area) and the anterior third of the petrous pyramid (Red trapezoid area). B, Petrous region DAVFs include SPS DAVFs (No. 1 blue area), IPS DAVFs (No. 2 green area), petrous apex DAVFs (No. 3 yellow area), upper clival DAVFs (No. 4 red area), and upper clival epidural-osseous DAVFs (No. 4 red area). C, The arterial supply of the lateral petroclival region includes the MMA (green circle), the AphA (red circle), and the OA (blue circle). D, The arteries supplying the petrous apex/upper clivus region dura are show in the blue rectangle area, the main feeding arteries are derived from the meningeal branches of the ICA. **Abbreviations:** AphA, ascending pharyngeal artery; DAVF, dural arteriovenous fistula; ICA, internal carotid artery; IPS, inferior petrosal sinus; MMA, the middle meningeal artery; OA, occipital artery; SPS, superior petrosal sinus.

Intracranial dural arteriovenous fistulas (DAVFs) are abnormal connections between the dural arteries and dural venous sinuses or leptomeningeal veins 1. DAVFs mostly involve the regions of the transverse, sigmoid, and cavernous sinuses. In rare circumstances, DAVFs could also be located at the petroclival region 2. The petroclival region refers to the junction of the petrosal and occipital bones and is mainly confined to the upper clivus and the anterior third of the petrous pyramid in front of the internal acoustic meatus 3-5. The petroclival region is illustrated in Figure 1A.

Petroclival region DAVFs have a variety of potential anatomical configurations and are defined by the involved venous sinus and plexus overlying the bony structures 6. Petroclival region DAVFs are difficult to treat. Surgical removal often carries the risk of compromising the important neighboring neurovascular structures 7. Although stereotactic radiosurgery is effective for intracranial DAVFs, it is not suitable for petroclival DAVFs. The DAVFs of petrosal region often present with hemorrhage and are at higher risk for rebleeding. Besides, the latent effective period is too long 8.

Currently, endovascular treatment (EVT) has become a first-line option for petroclival region DAVFs both for its effectiveness and low invasiveness 9. However, EVT is not a one-size-fits-all solution for petroclival region DAVFs. In most of the cases, there are multiple arterial feeders, and no main sinus drainage, which could provide interventional venous route, is involved 10. Considering their rarity and complexity, the petroclival region DAVFs still poses great challenge to both the neurosurgeons and neurointerventionalists. In order to further expound the EVT for petroclival region DAVFs, we performed a review of the relevant literature.

## Classification

The petroclival region mainly refers to the area of the upper clivus and the anterior third of the petrous pyramid in front of the internal acoustic meatus 3, 4. The dural and venous structures in this area mainly include the superior petrosal sinus (SPS), the inferior petrosal sinus (IPS), and the basilar plexus (BP) 11-13. Based on their positional and anatomical characteristics, the petroclival region DAVFs are classified into the SPS DAVF, IPS DAVF, petrous apex DAVF, upper clival DAVF, and upper clival epidural-osseous DAVF. Classification of the petroclival region DAVFs is illustrated in Figure 1B.

The SPS DAVF, also known as the petrous ridge DAVF, accounts for approximately 5% of all intracranial DAVFs 10, 14-16. The petrous apex DAVF often involves the free edge of the tentorium 7, 17. In Barnwell et al.'s study, the IPS DAVF accounts for approximately 6% of all intracranial DAVFs 18, 19. Due to their similarity in angioarchitecture, DAVFs involving the inferior petroclival vein (IPCV) are often regarded as IPS DAVFs 18, 20, 21.

The Upper clival DAVF and upper clival epidural-osseous DAVF are rare. The two distinct entities are distinguished from each other mainly based on their fistula locations: the former is on the dura, the latter is on the wall of the dilated venous pouch of the intraosseous veins 22-24.

## Angioarchitecture and grading

### Angioarchitecture

#### Feeding artery

The normal dural supplying arteries in the petroclival region can be divided in two groups: the lateral petroclival and the petrous apex/upper clivus groups 4, 25.

##### (i) Lateral petroclival region

For the DAVFs in the lateral petroclival region, including SPS and IPS DAVFs, the main feeding arteries originate from the meningeal branches of the external carotid artery (ECA), such as the ascending pharyngeal artery (AphA), the occipital artery (OA), and the middle meningeal artery (MMA) 10, 14, 16, 18, 21, 26-28. In rare cases, the meningeal branches of the internal carotid artery (ICA)and vertebral artery (VA) can supply the lateral petroclival region DAVFs 10, 15, 16, 18, 21, 29. In addition, the superficial temporal artery and the meningeal branches of the maxillary artery can also be involved in IPS DAVFs 18, 19.

More specifically, the jugular branch and hypoglossal branches of the AphA supplies the lateral wall of the IPS 30. The mastoid branch of the OA supplies the dura over the lateral portion of the posterior petrosal bone 31. The petrosal branch and the posterior division of the MMA supplies the dura over the insertion of the tentorium along the petrous ridge and the SPS 25, 32. The main arterial supply pattern to the lateral petroclival region dura is shown in Figure 1C.

##### (ii) Petrous apex/upper clivus region

For DAVFs in this region, the main feeding arteries are derived from the meningeal branches of the ICA 7, 15, 17, 22, 33, 34. The meningeal arteries of the ICA include the recurrent artery of the foramen lacerum, medial tentorial artery, lateral tentorial artery, dorsal meningeal artery, medial clival artery, and inferolateral trunk 25, 35, 36. The meningeal branches of the maxillary artery can also be involved 7, 15, 22. The arterial supply pattern to the petrous apex/upper clivus region dura is shown in Figure 1D.

Under rare circumstances, the anterior meningeal artery of the VA, the subarcuate artery of the anterior inferior cerebellar artery, and the tentorial branch of the posterior cerebral artery can also provide blood supply to the DAVFs in this region 23, 25, 37-39. For the clival epidural-osseous DAVFs, in addition to the aforementioned feeding arteries, the posterior auricular artery, internal maxillary artery, superficial temporal artery, and the muscular-dural and the ascending cervical branches of the VA can also be involved 24.

In general, the feeding arteries of petroclival region DAVFs originate from the ipsilateral side, and occasionally they might originate from both the ipsilateral and contralateral sides 18. In the condition of DAVFs, the blood-supplying range of the meningeal branches of the ECA, ICA, and VA becomes wider than in normal conditions. And these arteries also become unusually enlarged 21, 22.

#### Venous drainage

##### (i) Normal venous drainage structure

In the petroclival region, the posterior part of the cavernous sinus (CS), the SPS, the IPS, and the BP converge to form the petroclival venous confluence (PVC) 11-13. The superior petrosal vein (SPV) empties into the SPS 40. The BP is located between the periosteal and meningeal layers of the clivus and communicates with the CS, SPS, and IPS 41, 42. The IPS drains venous blood from the PVC and the BP then empties into the junction of the sigmoid sinus and the superior jugular bulb 43.

##### (ii) DAVF venous drainage patterns

Different types of petroclival region DAVFs have different venous drainage patterns. The SPS DAVF usually drains into the SPV and its tributaries, and occasionally the CS can act as the venous drainage route 16, 26, 29, 44, 45. Petrous apex DAVF mainly drains supratentorially into the Vein of Galen, the internal cerebral vein, and the straight sinus 17, 33, 46. The IPS DAVF has a similar venous drainage to that of the CS DAVF. It often drains retrogradely into the CS via the IPS and then out through the superior ophthalmic vein, and sometimes there is some antegrade flow into the sigmoid sinus 18, 21, 27, 28.

The venous drainage of the upper clival DVAF is from the clival veins to the CS and the superior ophthalmic vein 22, 34, 47, 48. It can also drain to the IPS or even the cortical vein. The venous drainage of the intraosseous DAVF is often through the adjacent sinus, such as the IPS, the jugular bulb, and the internal jugular vein 23, 24.

**Figure 2:**
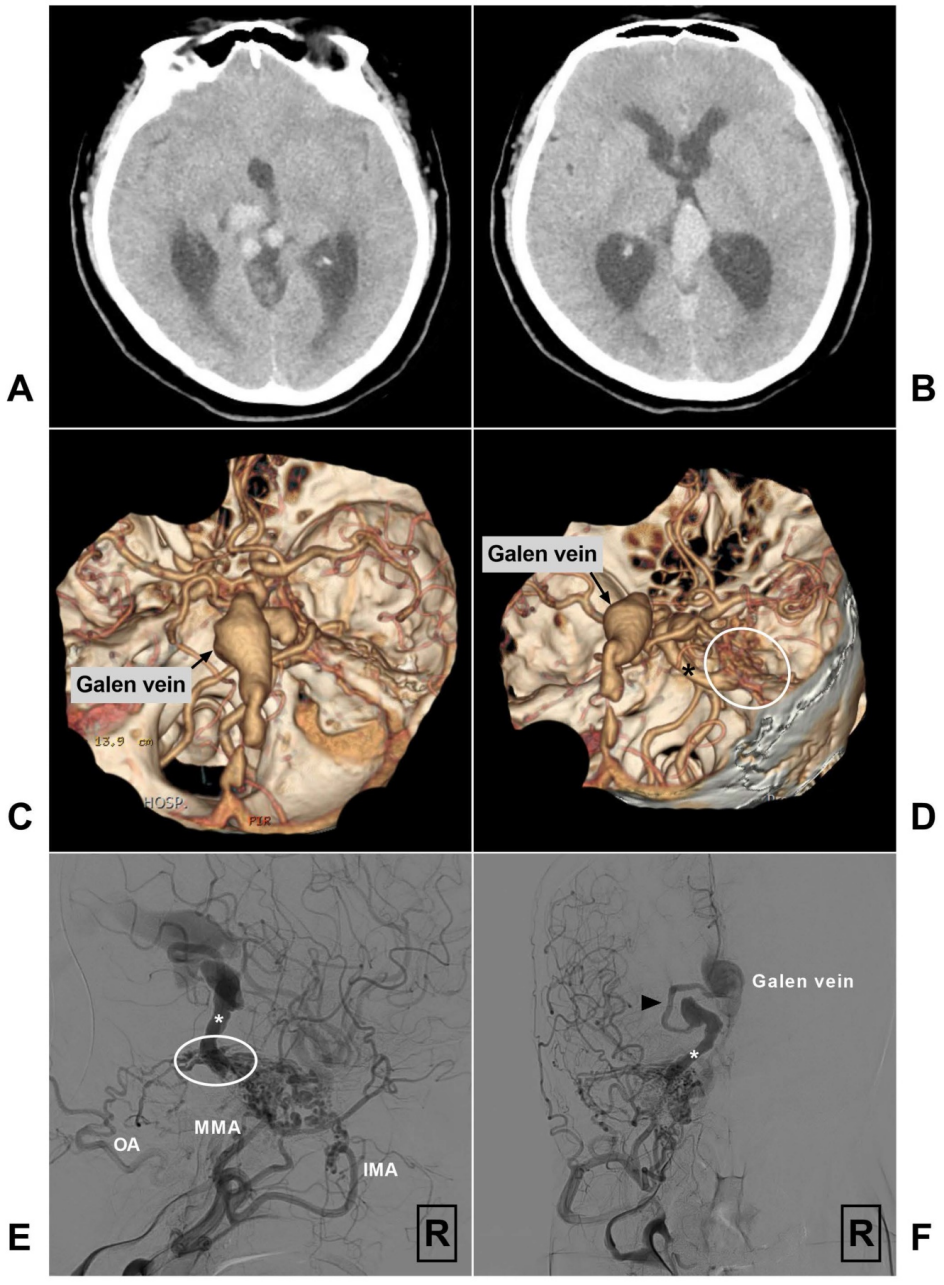
A-B, Head CT shows quadrigeminal cistern venous structure leading to obstructive hydrocephalus. C-D, CTA reveals a petroclival DAVF (encircled area) with enlarged petrosal vein (asterisk) and the Vein of Galen. E-F, Angiogram of the right ECA in lateral (E) and anteroposterior (F) views shows a SPS DAVF (encircled area) supplied by the IMA, MMA, and OA and drained retrogradely via the petrosal vein (asterisk) and pre-brainstem vein (arrow head) to the Vein of Galen. **Abbreviations:** CT, computed tomography; CTA, computed tomography angiography; DAVF, dural arteriovenous fistula; ECA, external carotid artery; IMA, internal maxillary artery; MMA, middle meningeal artery; OA, occipital artery, R, right; SPS: superior petrosal sinus.

In addition, venous outflow obstruction is common in petroclival region DAVFs, contributing to the arterialization of the thin-walled pial veins, which increases the venous pressure, promotes the formation of venous ectasia, or leads to venous infarction 15-19, 21, 23, 26, 49, 50. As illustrated in Fig 2, the enlarged petrosal vein and the Vein of Galen even lead to obstructive hydrocephalus. In rare circumstances, the venous drainage can even flow retrogradely into the spinal veins via the mesencephalic venous connections 14, 17, 33, 45, 51.

#### Fistula point

The fistula point of SPS DAVF is often located at the connection of the SPV and the SPS 10, 14, 16. The fistula point of petrous apex DAVF is located at the dura mater of the petrous apex or the free edge of the tentorium 7, 17. The fistula point of IPS DAVF is often close to the jugular bulb 27. The fistula point of the upper clival DAVF is at the clival dura mater on the clival venous plexus 22. The fistula point of intraosseous DAVF is on the wall of the dilated venous pouch of the intraosseous diploic veins or the transosseous emissary veins 23. The petroclival region DAVFs can be divided into the high-flow and low-flow types. The high-flow fistulas usually result from arterial overflow, whereas the low-flow fistulas may be caused by high venous pressure 46.

Angioarchitecture of the petroclival region DAVFs is summarized in Table 1.

### Grading

SPS and petrous apex DAVFs often have retrograde pial venous drainage, deep cerebral venous drainage, and venous ectasia. Hence, they are often categorized as types III or IV according to the Cognard's classification and are prone to have aggressive clinical courses 7, 10, 15, 52. IPS and upper clival DAVFs often drain antegradely into the venous sinuses, and they are prone to have lower Cognard's grades 18, 21, 23, 27, 28. The clival epidural-osseous DAVF often drains into the adjacent sinuses, so its Cognard's grade belongs to types I and II in most cases 24.

## Outline of EVT

Most petroclival region DAVFs should be treated due to their high Cognard's grades, especially for those with venous ectasia of alarming sizes 10. Currently, EVT has become a first-line option for most intracranial DAVFs, which includes transarterial embolization (TAE) and transvenous embolization (TVE) 53. The selection of TAE or TVE should be determined in a case by case approach, which is mainly based on the individual anatomic characteristics 48. The goal of EVT is complete obliteration of the lesion 54. To achieve definite obliteration via EVT, the embolic material has to plug the arteriovenous communication or seal the lumen of the draining venous structure 46.

Complete embolization is necessary. Though partial EVT can result in marked decrease in flow and venous hypertension, the risk of rebleeding remains unchanged 2. Moreover, with mere occlusion of feeding arteries, the DAVFs are likely to recruit new feeders, even after a previous complete angiographic cure 52. Currently, as a result of the development of new techniques and materials, particularly with the use of Onyx (Medtronic Neurovascular, Minneapolis, MN), TAE has become a more effective option and represents the first-line option for many DAVFs 10, 14. However, in some cases, TAE alone may be unsuccessful in obliterating the petroclival region DAVFs, because the feeding arteries are too tiny to permit super-selective embolization 10.

Currently, TVE is useful for intracranial DAVFs involving the major dural sinuses 22, 55. However, TVE for petroclival region DAVFs is difficult because the initial venous drainage mainly flows through the pial veins 15, 16. Therefore, TVE could only be tried in limited cases 10. For some intracranial DAVFs, treatment with combined TAE and TVE is superior 56. However, for the petroclival region DAVFs, the combined TAE and TVE approach is unnecessary in most cases. Of note, TVE after unsuccessful TAE or TAE after unsuccessful TVE does not belong to the combined treatment.

In summary, EVT cannot be readily used for all petroclival region DAVFs. When the arterial feeders are large or the DAVFs are adjacent to the venous sinuses, the success rate is high.

## Transarterial embolization

TAE is difficult for petroclival region DAVFs and can only be performed in selected cases with large and straight arterial feeders 21. During TAE, careful evaluation of the DAVF structure and dynamic blood flow change with 3-dimensional angiogram is necessary. Advancing the microcatheter more closely to the fistula site can increase the success rate and reduce the risk of inadvertent embolization of the non-targeted vessel 29.

The SPS, petrous apex, and upper clival DAVFs can be treated with TAE alone 7, 22, 33. Of note, using Onyx can result in higher cure rate 17, 22. Furthermore, the advance in improved flexibility, trackability, and shape retention of microcatheter design and the improvement of guidewire technology have permitted super-selective catheterization of more and more previously inaccessible arterial feeders 15. For most of the intracranial DAVFs, MMA is the best route during TAE, and there is no exception for petroclival region DAVFs 57.

### Transarterial path

For SPS and petrous apex DAVFs, the petrosquamous branch of the MMA is an effective transarterial path 10, 14, 21, 33, 45. For instance, Alleyne* et al*. reported an SPS DAVF with TAE 26. The microcatheter was advanced to the most distal branch of the posterior MMA and the SPS was completely occluded with n-butyl cyanoacrylate (NBCA).

In addition to the MMA, some other arteries can also be used, such as the mastoid branch of the OA, the neuromeningeal trunk of the AphA, and the anterior meningeal artery of the VA 10, 22, 45. For instance, the upper clival DAVF can be completely embolized via the clival branch of the AphA 22. However, TAE via other feeders than the MMA means a lower success rate and more risks 10, 22, 45.

### Onyx and NBCA

Onyx is widely used in TAE for its strong ability in penetrating the shunt and occluding the draining vein 22. However, for the embolization of petroclival region DAVFs, Onyx is associated with higher risk of complications, because the Onyx would partially reflux during pushing forward 58, 59. In contrast, NBCA tends to move forward during TAE and does not reflux 10, 45, 46. However, NBCA may fail to penetrate deeply enough to completely occlude the DAVFs, because its polymerization is unpredictable and its adhesive properties place the patient at the risk of permanently retaining the microcatheter 14, 28, 60-62. Therefore, the choosing of a specific embolic agent should depend on the specific angioarchitecture 22, 29, 63.

**Figure 3:**
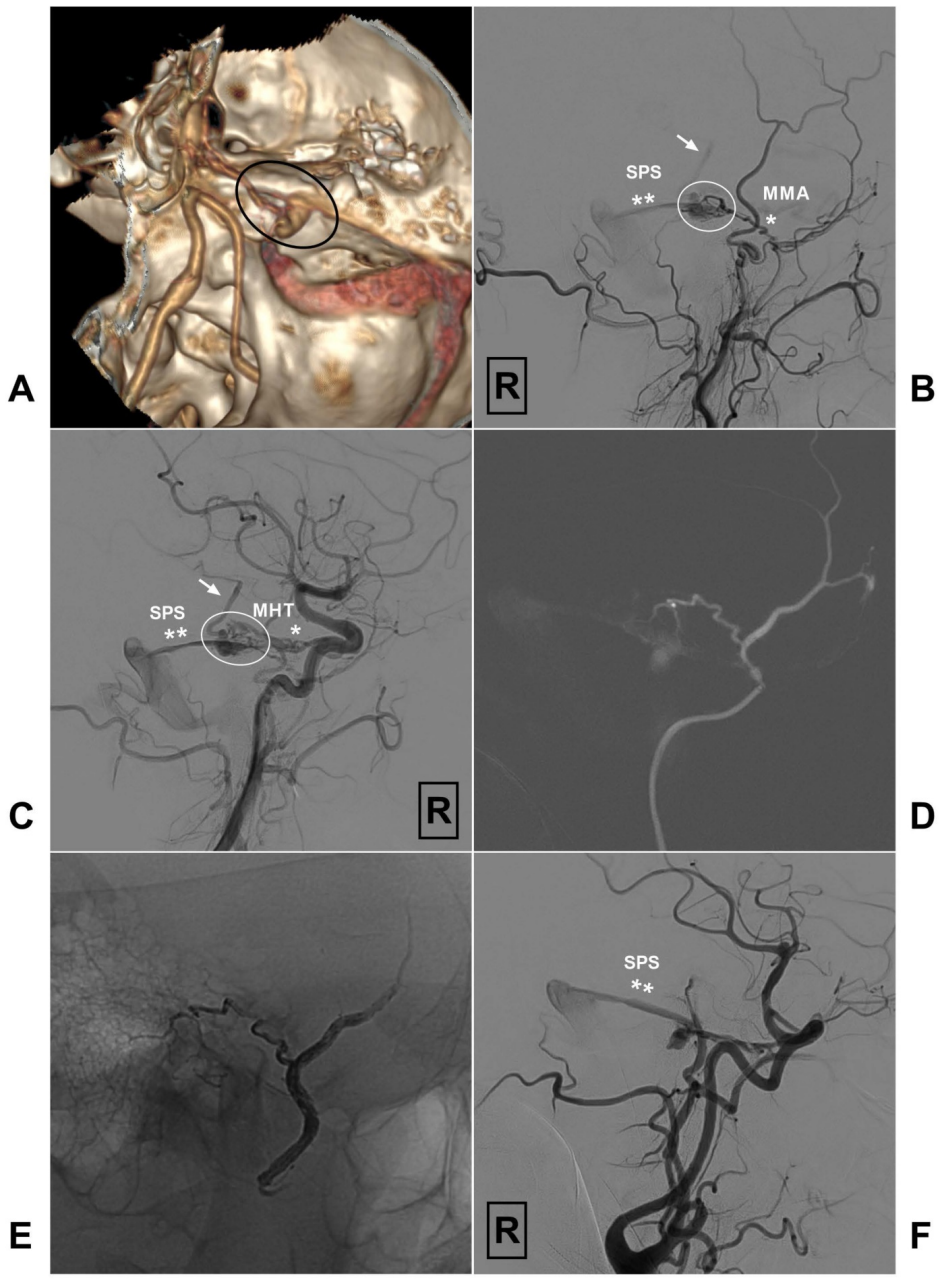
A, CTA reveals a petroclival DAVF (encircled area). B, Angiogram of the right ECA in lateral view shows the DAVF (encircled area) is supplied by the MMA (single asterisk) and drains to the SPS (double asterisks). Besides, retrograde venous drainage (arrow) to the brainstem is also noted. C, Angiogram of the right ICA in right anterior oblique view shows the DAVF (encircled area) also receives blood supply from the MHT (single asterisk) and drains to the SPS (double asterisks). Retrograde venous drainage (arrow) to the brainstem is also noted. D, Super-selective angiogram of the right MMA shows the branch supplying the DAVF is tortuous and enlarged. E, Onyx could not penetrate the fistula point during transarterial embolization of the DAVF via the MMA. F, Angiogram of the ICA shows the DAVF still has residual venous drainage via SPS (double asterisks). **Abbreviations:** CTA, computed tomography angiography; DAVF, dural arteriovenous fistula; ECA, external carotid artery; ICA, internal carotid artery; MHT, meningohypophyseal trunk; MMA, the middle meningeal artery; R, right; SPS: superior petrosal sinus.

**Figure 4:**
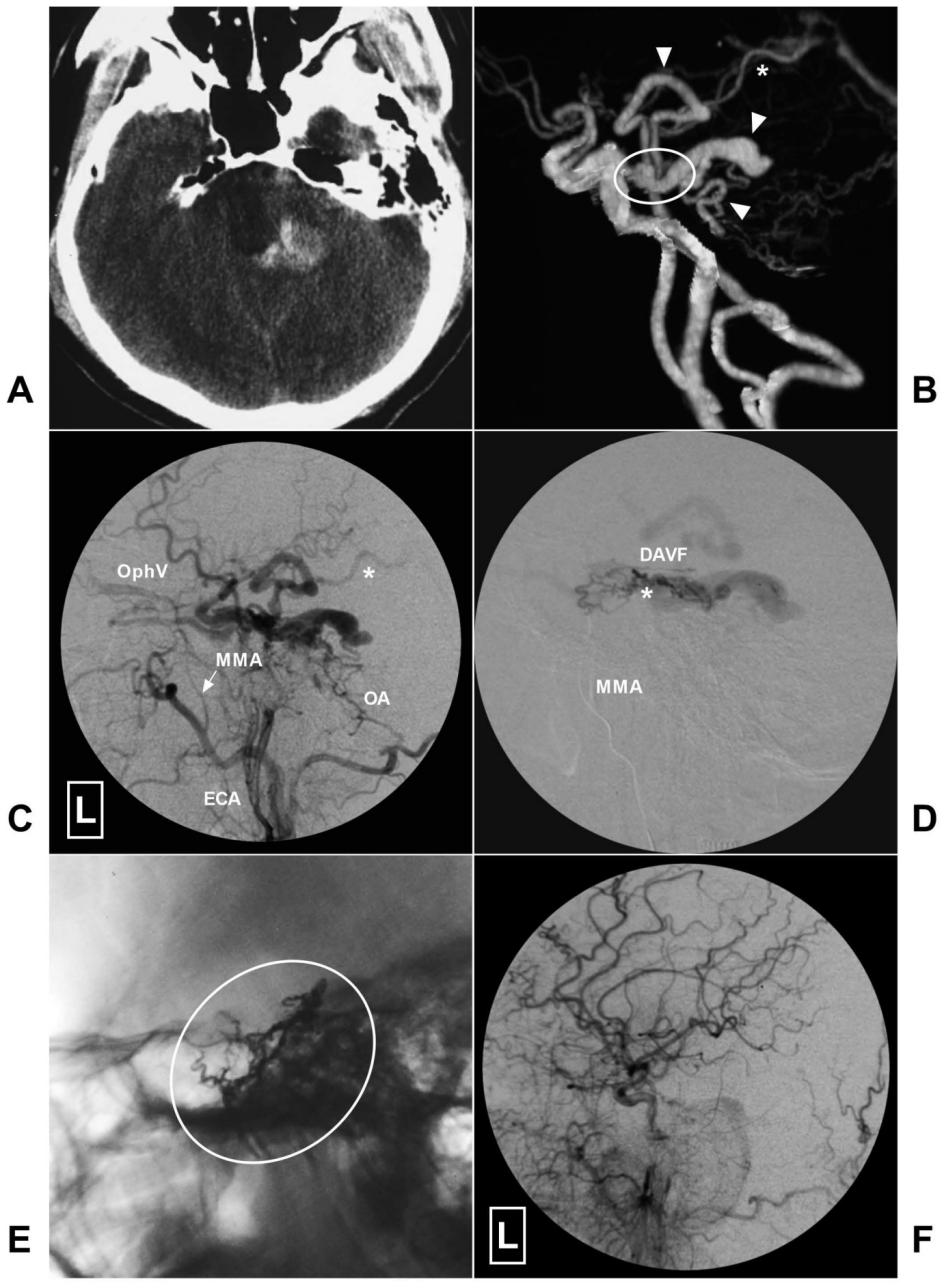
A, CT shows hemorrhage at the left side posterior to the brainstem. B, CTA reveals dilated venous structure (arrow heads) around the brainstem draining to the Vein of Galen (asterisk) indicating a petroclival DAVF (encircled area). C, Angiogram of the left ECA in lateral view shows the DAVF is mainly fed by the MMA (arrow) and OA and drains to the peri-brainstem and ophthalmic vein. Asterisk denotes the vein drains to the Vein of Galen. D, Super-selective angiogram via microcatheter shows the DAVF. Asterisk denotes the fistula point. E, X-ray of the cranium reveals Onyx casting (encircled area) through the MMA. F, Angiogram of the left CCA in lateral view after EVT shows the DAVF is completely obliterated. **Abbreviations:** CCA, common carotid artery; CT, computed tomography; CTA, computed tomography angiography; DAVF, dural arteriovenous fistula; ECA, external carotid artery; L, left; MMA, middle meningeal artery; OA, occipital artery; OphV, ophthalmic vein.

In Fig 3, we present an unsuccessful case with TAE. An illustrative case of petroclival DAVF treated with successful TAE via the MMA is also presented in Fig 4.

## Transvenous embolization

For petroclival region DAVFs, TVE may be a good option in some cases. SPS and IPS DAVFs may be good candidates because these DAVFs are adjacent to the SPS and IPS. For the TVE of SPS and IPS DAVFs, the detachable coils are effective in blocking the initial part of the draining vein 15, 16, 18, 28. Even after mere occlusion of the leptomeningeal vein to reduce venous pressure, the outflow of residual DAVF could be stopped, leading to subsequent venous thrombosis inside the dura 17, 45.

For instance, according to Seong* et al*.'s report, an SPS DAVF was completely occluded after a coil was pushed to the fistula site via the SPV 10. In another case reported by Gentric* et al*., an IPS DAVF was coiled via the IPS after percutaneous puncture of the superior ophthalmic vein 27. IPS DAVF can be occluded tranvenously even though the IPS is thrombosed 18.

Occasionally, for the upper clival DAVF, the draining clival venous structures can be embolized with coils via the IPS 48. However, when the DAVFs empty directly into the clival veins rather than into the adjacent sinuses, transvenous coiling without occlusion of the fistulous sites may be ineffective 22, 47. In intraosseous DAVF, the feeding arteries are centered on the wall of the dilated venous pouch that is located within the bone and communicate with the venous structures. Therefore, this type of DAVF could be completely occluded with TVE. For curative treatment, the venous pouch can be the target during endovascular treatment 23. Rarely, for SPS DAVF, TVE is possible through a patent occipital sinus 16. IPCV DAVF can also be treated with TVE 20.

However, for most petroclival region DAVFs, due to the absence of connection with the venous sinus, the occlusion of the downstream sinus, or significant venous stenosis near the junction of the draining vein and the sigmoid sinus, TVE is impossible 50. TVE through the fragile pial veins might result in premature rupture of the draining veins 15, 16.

**Figure 5:**
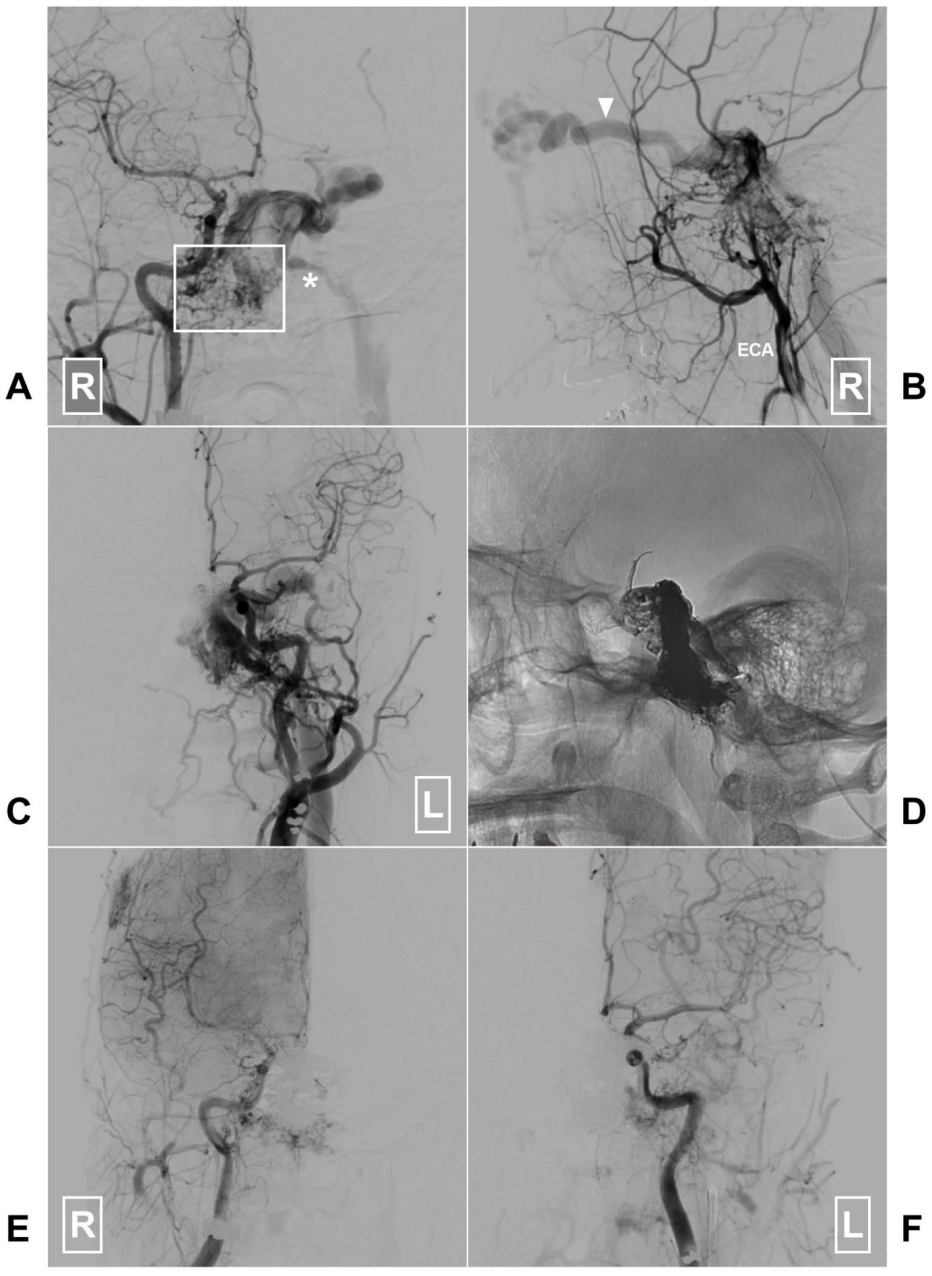
A, Angiogram of the right CCA in AP view reveals a clival DAVF (rectangle) draining to the contralateral IJV (asterisk) via the sigmoid sinus. B, Angiogram of the right ECA in lateral view shows there are multiple tiny feeding arteries. Arrow head denotes the ophthalmic vein drainage. C, Angiogram of the left CCA in AP view shows branches of the left ECA also supply the DAVF. D, X-ray of the cranium shows the Onyx casting after embolization of the through the left IJV. E-F, Angiogram of the right and left CCAs in AP view shows the DAVF is completely obliterated. **Abbreviations:** AP, anteroposterior; CCA, common carotid artery; DAVF, dural arteriovenous fistula; ECA, external carotid artery; IJV, internal jugular vein; L: left; R, right.

An illustrative case of petroclival DAVF treated with successful TVE is presented in Fig 5.

## Complications

### Cranial nerve palsy

The petroclival region contains some important cranial nerves. The petroclival region DAVFs often has intersecting arterial supply with these cranial nerves. Excessive reflux of liquid embolic agents during TAE of the petroclival region DAVFs could lead to cranial nerve palsy 7, 18, 22.

### Venous sinus thrombosis

After EVT of the petroclival region DAVFs, the associated sinus might experience subsequent thrombosis. For instance, in Barnwell* et al*.'s study, one patient developed extensive thrombosis of the ipsilateral transverse-sigmoid sinus and the jugular bulb 1 week after the embolization 18.

### Migration embolization

At the petroclival region, there are many dangerous anastomoses. Therefore, before TAE, careful preoperative evaluation of the super-selective angiogram is necessary to identify potential collaterals to the ICA and the VA. Migration embolization of the ICA and the VA should be avoided 18.

## Prognosis

In petroclival region DAVFs, if EVT can be performed successfully, a satisfactory outcome is anticipated 15, 33. However, only a certain proportion of the patients could be successfully treated with EVT. For instance, in Ng* et al*.'s study, only 4 of the 14 patients (22%) underwent successful TAE and TVE without adjunctive surgery 15. Compared with TAE, TVE can result in higher cure rate. In Barnwell* et al*.'s report, 5 of the 6 patients received successful TVE 18.

## Summary

Petroclival region DAVFs are rare and often have very complex anatomical structures. They can be further divided into the SPS, petrous apex, IPS, upper clival, and upper clival epidural-osseous DAVFs. Most petroclival region DAVFs should be treated due to their high Cognard grades.

Currently, EVT has become the first-line therapeutic option for petroclival region DAVFs. But not all the petroclival region DAVFs could be cured with EVT. When the arterial feeders are large or the DAVF is adjacent to the venous sinus, the success rate may be higher.

In petroclival region DAVFs, if EVT can be performed successfully, satisfactory outcomes can be anticipated. However, there are some inadvertent complications, which include cranial nerve palsy, subsequent sinus thrombosis, and migration embolization of the ICA and the VA.

## Figures and Tables

**Table 1:** Angioarchitecture of petroclival region DAVFs

Types	Main feeding artery	Fistula point	Main venous drainage
SPS DAVF	AphA, OA, and MMA	Dura at the connection between the SPV and the SPS	SPV and its tributaries, and occasionally the CS
IPS DAVF	AphA, OA, and MMA	Dura close to the right jugular bulb	Retrograde flow through the IPS to the CS, and sometimes antegrade flow to the sigmoid sinus
Petrous apex DAVF	Meningeal branches of the ICA	Petrous apex dura or the free edge of the tentorium	Vein of Galen, the internal cerebral vein, and the straight sinus
Upper clival DAVF	Meningeal branches of the ICA	Clival dura on the clival venous plexus	CS or to the IPS and even to the cortical vein
Upper clival epidural-osseous DAVF	Meningeal branches of the ICA	Wall of the intraosseous diploic veins or the transosseous emissary veins	IPS, jugular bulb, and internal jugular vein

**Abbreviations:** AphA, ascending pharyngeal artery; CS, cavernous sinus; DAVF, dural arteriovenous fistula; ICA, internal carotid artery; IPS, inferior petrosal sinus; MMA, the middle meningeal artery; OA, occipital artery; SPS, superior petrosal sinus; SPV, superior petrosal vein.
